# A flame retardant-hardener for epoxy resins: Synthesis, structural, and DFT studies of the [Cu(H_2_NC_2_H_4_NH_2_)_2_(H_2_O)Cl]Cl complex

**DOI:** 10.3906/kim-2106-9

**Published:** 2021-08-11

**Authors:** Borys MYKHALICHKO, Helen LAVRENYUK, Oleg MYKHALICHKO

**Affiliations:** 1 Department of Physics and Chemistry of Combustion, L’viv State University of Life Safety, L’viv, Ukraine; 2 Limited Liability Company “FUCHS Mastyla Ukraina”, L’viv, Ukraine

**Keywords:** Copper(II) chelate complexes, synthesis, X-ray crystal structure determination, IR spectroscopy, DFT calculation

## Abstract

The aqua-*bis*(ethylenediamine)-chloro-copper(II) chloride complex, [Cu(*en*)_2_(H_2_O)(Cl)]Cl (**1**), was synthesized by direct interaction of CuCl_2_×2H_2_O with *pepa* (*pepa* is a polyethylenepolyamine containing ethylenediamine (*en*)). The crystalline complex **1** was characterized by IR spectra and structurally studied. Crystals of this complex consist of the [Cu(*en*)_2_(H_2_O)(Cl)]^+^ discrete cations, whose Cu^2+^ ion is chelated by two *en* molecules. The Cu(II) coordination polyhedron has the shape of an elongated square bipyramid, in which four nitrogen atoms of the –NH_2_ groups of *en* molecules form the base of this bipyramid, and the oxygen atom of the water molecule and Cl^−^ are in its apical positions. Three-dimensional framework **1** is formed due to hydrogen bonds O–H…Cl and N–H…Cl. The Cu(II)–(H_2_NC_2_H_4_NH_2_) chelation was studied by DFT using the 6-31G* basis set. The calculated electron-stereochemical parameters are in good agreement with the ability of complex **1** to simultaneously be a flame retardant and a curing agent of epoxy resins.

## 1. Introduction

Recently, chelate complexes [1] of many *d*-metals with polyamines [2] due to their thermal stabilization [3–8] caused by the chelating effect are widely used as flame retardants and hardeners of epoxy resins [9–15]. Among the great number of polydentate ligands, ethylenediamine (*en*) is known to be used as an efficient curing agent to produce epoxy-polymer composites. In turn, high-performance materials based on epoxy resins are one of the most demanded classes of polymers used in industry today, from simple two-component adhesives to high-tech applications [16]. However, the epoxy-polymer composites are combustible materials, which prevents their wider use. Nevertheless, their combustibility can be significantly reduced if polyamine complexes of transition metals are used as a modifier for the production of epoxy-amine composites. In this regard, the chelate complex of incombustible copper(II) chloride with *en* is of particular interest [17]. To better understand how chelate complexes containing a polyamine ligand bonded to a central metal atom can affect the efficiency of epoxy-amine polymerization and increase the flame retardant properties of copper(II)-containing epoxy-amine composites, reliable information is needed on the crystal structure of chelate complexes. Unfortunately, the data on the crystal structure of the chelate complex of copper(II) chloride with ethylenediamine in [17] turned out to be inaccurate.

This study reports the interaction of polyethylenepolyamine (*pepa*) with copper(II) chloride to form the crystalline complex [Cu(*en*)_2_(H_2_O)(Cl)]Cl (**1**) (*en* is one of the components of *pepa*), the crystal structure of which was determined more precisely, and its electron-stereochemical parameters were calculated by DFT.

## 2. Experimental part

### 2.1. Synthesis

The crystal complex [Cu(*en*)_2_(H_2_O)(Cl)]Cl (**1**) was obtained by direct interaction of copper(II) chloride with *pepa* (*pepa* = H_2_N[–C_2_H_4_NH–]_n_H containing *en* = NH_2_C_2_H_4_NH_2_). Crystalline CuCl_2_×2H_2_O (1.7 g, 0.01 mol) was placed in a porcelain mortar, an excess of *pepa* was added, and the resulting mixture was triturated until a homogeneous liquid suspension of dark blue color appeared. The contents were left for several days at room temperature until crystalline phase **1** appeared according to the reaction (1).


(1) $${\text{CuCl}}_{2}\times 2{\text{H}}_{2}\text{O}+2\;{\text{NH}}_{2}{\text{C}}_{2}{\text{H}}_{4}{\text{NH}}_{2}\to [\text{Cu}{\left({\text{NH}}_{2}{\text{C}}_{2}{\text{H}}_{4}{\text{NH}}_{2}\right)}_{2}({\text{H}}_{2}\text{O})(\text{Cl})]\text{Cl}+{\text{H}}_{2}\text{O}$$

### 2.2. Single crystal structure determination

Diffraction data for the single crystal of complex **1** were collected on an ENRAF NONIUS CAD-4T diffractometer with Mo *K*_α_ radiation. Crystal data, data collection, and structure refinement details are summarized in Table 1. The crystal structure was solved and then refined by least squares method on *F*^2^ using the WinCSD, Version 4.19 software package [18]. The final refinement was done by ShelXL software [19] with the following graphical user interface of OLEX^2^ [20]. H atoms of –NH_2_ groups were derived from difference Fourier maps and refined with *U*_iso_(H) = 1.2 *U*_eq_(N). Water H atoms were also derived from difference Fourier maps and refined isotropically with O–H fixed distance. The other H atoms were refined in ideal positions (riding model), with C–H = 0.97 (methylene) and with *U*_iso_(H) = 1.2*U*_eq_(C). The position and thermal parameters of atoms for complex **1** is given in Table S1, Suppl. Info. Selected bond lengths and angles for complex **1** are presented in Table 2. Figures of the crystal structure **1** were drawn using DIAMOND 3.1 software.

### 2.3. IR spectroscopy

IR absorption spectra were recorded for crystalline complex **1** pressed in spectroscopically pure KBr pellet and a liquid sample of *pepa* using a KBr cuvette using the Perkin Elmer SpectrumTwo FTIR spectrometer (the spectral range from 4000 to 500 cm^−1^ with a resolution of 2 cm^−1^).

H_2_N[–C_2_H_4_NH–]*_n_*H: (n_N–H_ 3368, 3246, 3230, 3214, 3198); (n_C–H_ 2934, 2910, 2896, 2786); (d_N–H_ 1604, 1596); (d_C–H_ 1458, 1352); (N–C 1298, 1134, 1122, 1064, 1034); (962···752).

[Cu(NH_2_C_2_H_4_NH_2_)_2_(H_2_O)(Cl)]Cl: (3434–3390); (n_N–H (coord)_ 3350, 3320, 3280, 3264); (n_C–H_ 2946, 2920, 2876); (d_N–H (coord)_ 1624, 1584); (1564); (d_C–H_ 1476, 1450, 1388); (N–C (coord) 1326, 1286, 1252, 1112, 1026, 1014); (982, 890, 656, 618, 582).

### 2.4. Testing for flammability

The temperatures of ignition (*T*_ign_) and self-ignition (*T*_self-ign_) for *en* and [Cu(*en*)_2_(H_2_O)(Cl)]Cl were measured using the TF apparatus for temperature tests according to all-Union State Standard 12.1.044-89 [21], described in detail in Data S2, Suppl. Info. There were three measurements for each type of specimens; the resulting values were averaged. The values of *T*_ign_ and *T*_self-ign_ for *en* were 45(1) °C and 380(1) °C, respectively, while the crystalline complex **1** did not ignite or self-ignite even when the temperature in a reaction chamber reached 450(1) °C or 600(1) °C, respectively.

### 2.5. DFT study

Quantum-chemical modeling of chelating processes in the *pepa* – CuCl_2_·H_2_O system was carried out by the DFT method. The restricted formalism of B3LYP method with a 6-31G* orbital basis set was performed using the HyperChem program version 8.0.6. The [Cu(H_2_O)_2_Cl_2_] and [Cu(*en*)_2_(H_2_O)(Cl)]Cl discrete clusters, as well as a free molecule of *en*, were constructed using the crystallographic data of CuCl_2_·2H_2_O [22] and **1**. The charge density distribution on atoms was calculated with no geometrical optimization of the [Cu(H_2_O)_2_Cl_2_], and [Cu(*en*)_2_(H_2_O)(Cl)]Cl structural fragments, as well as the *en* molecule. The calculations were carried out under the assumption that the clusters and the ligand molecule are in vacuum as isolated particles.

## 3. Results and discussion

### 3.1. Structural features on Cu(II)–(*en*) binding in a κ^2^ mode

**Figure 1:**
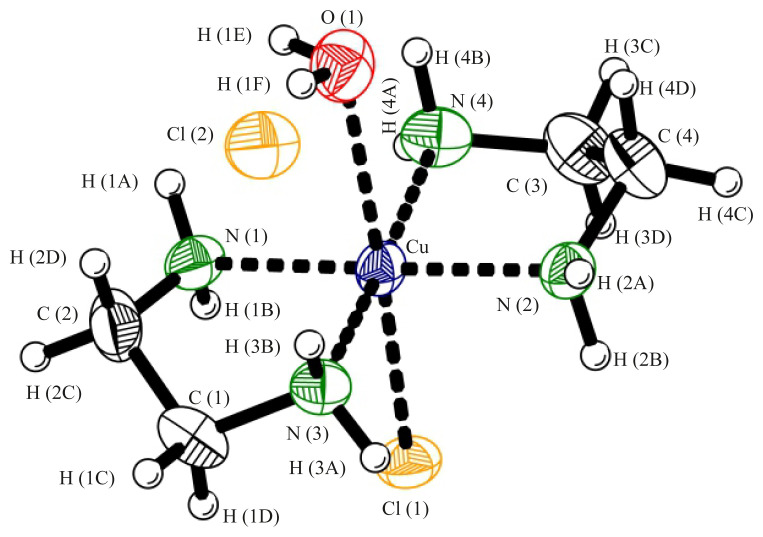
Crystallographically independent fragment of the crystal structure **1** with numbered atoms. Thermal ellipsoids are displayed at the 50% probability level for nonhydrogen atoms.

The ability of the amino-group to easily coordinate with copper(II) salts by donor-acceptor type, as well as the terminal arrangement of the amino-groups in the *en* molecule make this bidentate ligand an applicable chelating agent to form the [Cu(H_2_NC_2_H_4_NH_2_)_2_(H_2_O)(Cl)]Cl complex. Figure 1 shows the structural unit of complex **1**, in which the [Cu(*en*)_2_(H_2_O)(Cl)]^+^ discrete complex cation is formed, containing Cu^2+^ ions chelated by two bidentate molecules of *en*. The Cu(II)–(*en*) bonding in a κ^2^ mode, in turn, causes the deformation of the initially quadrilateral coordination core of Cu(II) generated by four N atoms of two *en* (see Table 2).

**Figure 2:**
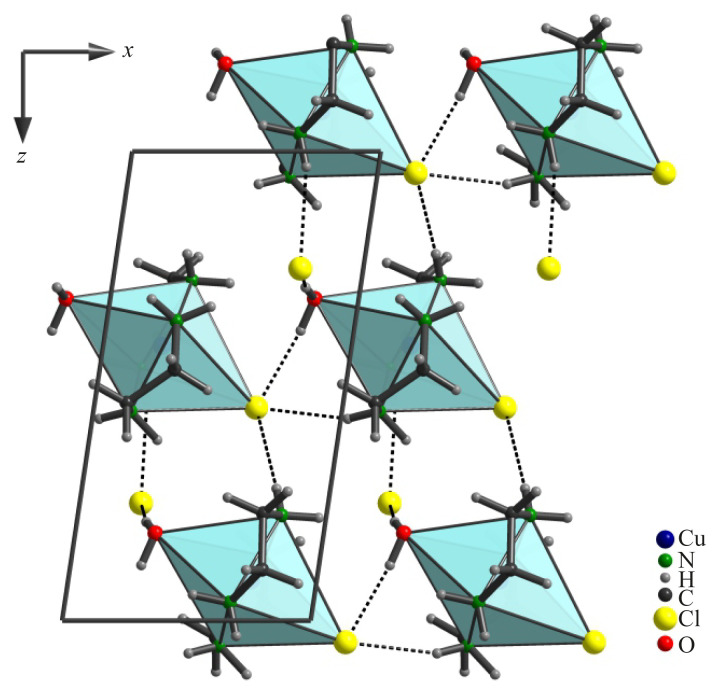
*XZ* plane projection of the crystal structure **1**. The coordination polyhedron of Cu(II) is highlighted.

Nevertheless, the Cu^2+^ ion in complex **1** is hexa-coordinated. In addition to four nitrogen atoms, the coordination core of Cu(II) also includes an oxygen atom of the H_2_O molecule and one Cl^−^ ion. Thus, the coordination environment of Cu(II) is an elongated square bipyramid (Figure 2), in which the N(1), N(2), N(3), and N(4) atoms form the basis of this bipyramid, while the O and Cl(1) atoms occupy two opposite apical positions at distances of 2.659(4) and 2.831(3) Å for Cu–O and Cu–Cl(1), respectively (the Jahn-Teller effect [23]). It should be noted here that in the crystal structure of the complex under consideration, studied by the X-ray photographic method in 1967 [17], the same Cu–O distance is 1.9(2) Å due to the incorrect determination of the positional parameters for the oxygen atom. Another chlorine atom, Cl(2), is an outer-sphere anion. Thus, this outer-sphere Cl^−^ anion is incorporated into the crystal framework **1**, crosslinking the [Cu(*en*)_2_(H_2_O)(Cl)]^+^ complex cations by hydrogen bonds N–H…Cl and O–H…Cl [24] ((N)H(4A)...Cl(2) and (O)H(1F)...Cl(2) distances are 2.55(9) and 2.22(3) Å, respectively), which additionally stabilizes the crystal structure of complex **1**.

The obtained structural data for complex **1** are in a good agreement with the observed shift in the vibration frequencies of N–H bonds in the IR spectra upon coordination with the metal atom. In particular, the CuCl_2_–(NH_2_C_2_H_4_NH_2_) chelation is reflected in the vibration frequency of the N–H bonds, which corresponds to the values of Dn_N–H_ = 20 cm^−1^ and Dd_N–H_ = 34 cm^−1^ for stretching and bending of –NH_2_ groups, respectively.

### 3.2. Electron preconditions for chelate complex formation

**Figure 3:**
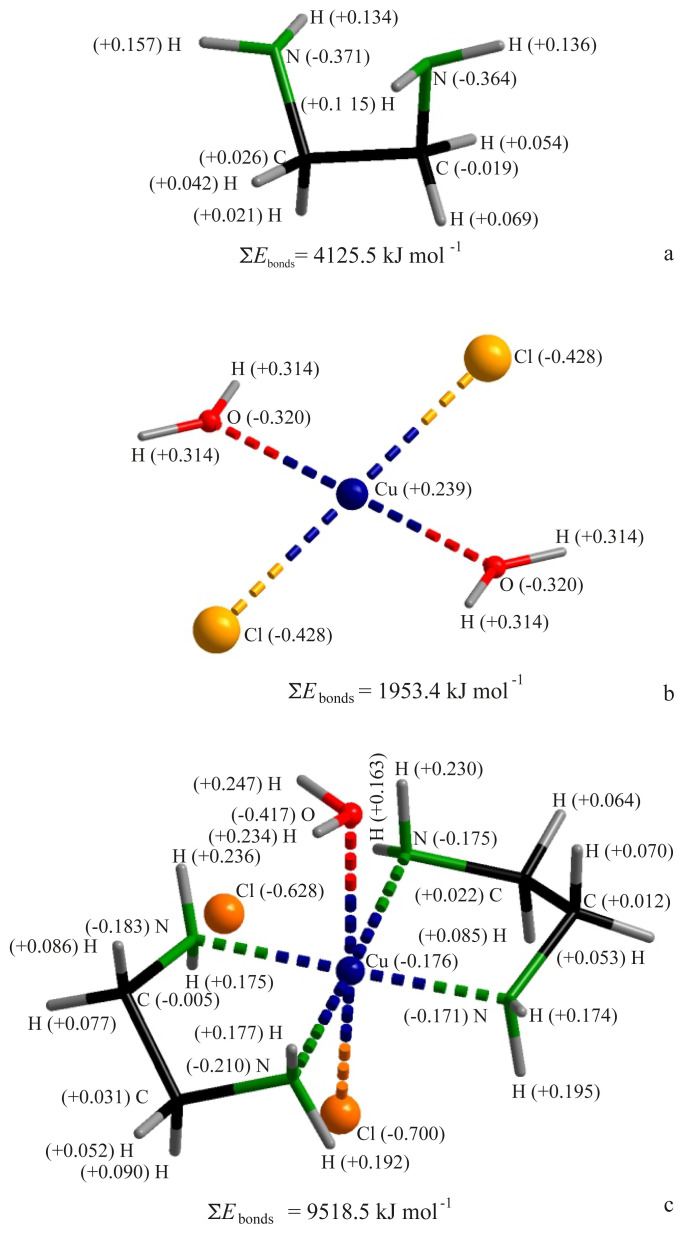
Charges (±d, ē) on atoms in *en* (*a*), [Cu(H_2_O)_2_Cl_2_] (*b*), and [Cu(*en*)_2_(H_2_O)Cl]Cl (*c*).

It is not less interesting to be considered the electron density distribution in the Cu(II) coordination core of complex **1**. Changes in the electronic parameters of the coordinated *en* molecule in comparison with its uncoordinated state are the result of chelation occurring in the *pepa* – CuCl_2_·2H_2_O system. The DFT study showed that in the Cu(II) coordination core, the electron density of N atoms is effectively shifted to the metal atom due to the chelating effect. The charge (±d, ē) on nitrogen atoms of –NH_2_ groups in an uncoordinated *en* molecule are −0.371, and −0.364 ē and the d value on the copper atom in CuCl_2_·2H_2_O is +0.239 ē (Figures 3a and 3b). The electron density on N atoms of –NH_2_ groups of *en* molecule noticeably decreases, owing to the formation of the [Cu(*en*)_2_(H_2_O)Cl]Cl chelate complex (the d values are −0.175, −0.171, −0.183, and −0.210 ē for coordinated nitrogen atoms). On the contrary, the electron density on the central copper atom is increased (in complex **1**, the d value for Cu atom is −0.176 ē) (Figure 3c).

### 3.3. Flame retardant-hardener properties of [Cu(*en*)_2_(H_2_O)Cl]Cl

**Figure 4:**
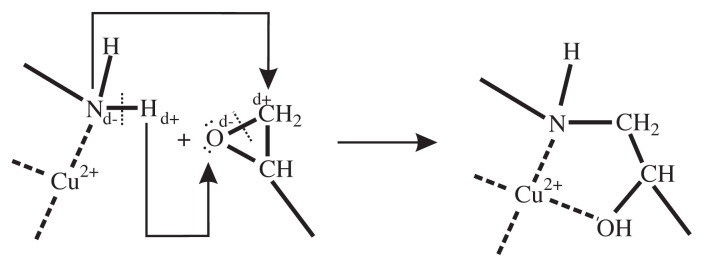
Scheme of the epoxy-curing by the flame retardant-hardener.

It can be expected that the observed electron density redistribution in the Cu(II) coordination core will have a positive effect on the epoxy-curing by the [Cu(*en*)_2_(H_2_O)Cl]Cl complex. Apparently, the Cu(II)–(*en*) chelating is able to cause polarization of N–H bonds, which results in an increase in the electrophilic ability of H atoms in amino groups. As a result, the positive charge on the H atoms in the coordinated amino-groups increases in comparison with the uncoordinated amino-groups (in complex **1**, the d values for H atoms of –NH_2_ groups are ranged from +0.163 to +0.236 ē) (see Figures 3a and 3c). All this facilitates the electrophilic addition of the H atom to the O atom of the oxirane ring as much as possible and, at the same time, enhances the ability of the N atoms to nucleophilic attack on the C atoms of epoxy groups (Figure 4). Thus, the analysis of charges on atoms of complex **1** clearly shows that *en* coordinated to Cu (II) is a more effective curing agent for epoxy resins than uncoordinated *en*.

On the other hand, the formation of a chelate complex is accompanied by the efficient binding of a combustible organic amine with a noncombustible inorganic salt. This interaction largely determines the thermal stability of the resulting chelate complex, which is able to act as a fire retardant hardener of epoxy resins in the epoxy-amine polymerization. Flammability tests have shown that *en* in the free state ignites at 45 °C. However, *en* being in the chelate complex **1** does not ignite at all. In other words, *en* as a combustible substance, after binding with CuCl_2_ and the formation of the [Cu(*en*)_2_(H_2_O)Cl]Cl complex, turns into a practically noncombustible substance. This fact can be explained by additional chemical bonds that form between *en* and CuCl_2_. DFT calculations of the energies of chemical bonds in a square-bipyramidal environment of Cu(II), carried out for the [Cu(*en*)_2_(H_2_O)Cl]Cl chelate complex, showed that the sum of the energies of four Cu–N bonds, one Cu–O bond, and one Cu–Cl bond is 338.1 kJ·mol^−1^. To break these bonds, it is necessary to use up a significant part of the thermal energy coming from the ignition source. In addition, for the ignition of *en*, a gas mixture of *en* and air must form above its surface. In this mixture, the saturated vapor concentration of *en* must exceed the lower limit of the concentration of flame propagation. However, even at temperature above 450 °C, the ignition of the [Cu(*en*)_2_(H_2_O)Cl]Cl complex was not observed. It should be noted that the previously studied chelate complex ethylenediamine-N,N′-diethylenetriamine-N,N′,N″-copper(II) hexafluorosilicate, [Cu(*en*)(*dien*)]SiF_6_ [6] containing *en* as a curing agent for epoxy resins and CuSiF_6_ as a flame retardant exhibits the properties of a flame retardant-hardener similar to complex **1**. Thermal analysis data performed for the [Cu(*en*)(*dien*)]SiF_6_ complex and its components (*pepa* and CuSiF_6_) showed that, in contrast to *pepa*, for which the total weight loss is observed in the temperature range from 20 °C to 170 °C, the [Cu(*en*)(*dien*)]SiF_6_ complex completely decomposes at a temperature of many times higher than 544 °C. This fact is explained by the ability of polyamine molecules to be firmly held in the complex by means of Cu(II)–N bonds. This chelation also makes it difficult for the polyamines to evaporate and ignite further. It is obvious that the main reason for the flame retardant influence of copper(II) salts on nitrogen-containing hydrocarbons is the complexing processes occurring in the polyamine–inorganic copper(II) salt system.

## 4. Conclusions

The chelate complex [Cu(*en*)_2_(H_2_O)Cl]Cl (**1**), which can be successfully used as a flame retardant-hardener of epoxy resins, was synthesized by direct interaction of CuCl_2_·2H_2_O with *pepa*. Crystalline complex **1** consists of discrete complex cations [Cu(*en*)_2_(H_2_O)Cl]^+^, whose Cu^2+^ ions are chelated by two *en* ligands. The complex cation is an elongated square bipyramid, the ligands of which are two bidentate *en* molecules, a water molecule, and a chloride ion. Hydrogen bonds N–H…Cl and O–H…Cl additionally stabilize crystal structure **1**. DFT analysis of electron structure **1** showed that *en* coordinated with Cu(II) is a more effective epoxy curing agent than *en* in the free state. The Cu(II)–*en* chelation is able to affect the flammability of the crystalline complex **1**. As a result, the combustible *en*, after bonding with copper(II) chloride, turns into a practically noncombustible substance. All this allows crystals **1** to be used as an effective flame retardant-hardener for epoxy resins.

**S1**. Position parameters of atoms and their thermal parameters for the [Cu(H_2_NC_2_H_4_NH_2_)_2_(H_2_O)Cl]Cl complex (**1**)

**S2**. Experimental determination details of the ignition and self-ignition temperatures of solids and materials.

**Table S1:** Position parameters of atoms and their thermal parameters for complex **1**

Atom	*x*	*y*	*z*	Ueq *, *Å2*

Cu	0.26440(9)	0.22821(3)	0.08533(4)	0.0304(2)
Cl(1)	−0.15275(17)	0.20728(7)	−0.04648(10)	0.0418(2)
Cl(2)	0.2537(2)	0.07007(9)	0.75006(11)	0.0537(3)
N(1)	0.3520(5)	0.2846(3)	−0.0534(2)	0.0377(7)
N(2)	0.1816(5)	0.1699(2)	0.2243(3)	0.0353(6)
N(3)	0.2200(5)	0.3529(2)	0.1384(3)	0.0360(6)
N(4)	0.3375(6)	0.1054(2)	0.0382(3)	0.0420(7)
C(1)	0.1988(8)	0.4087(3)	0.0353(5)	0.0465(9)
C(2)	0.3716(7)	0.3800(3)	−0.0357(4)	0.0492(9)
C(3)	0.2270(9)	0.0439(3)	0.1087(5)	0.0518(10)
C(4)	0.2558(9)	0.0784(3)	0.2285(5)	0.0509(10)
O	0.6775(6)	0.2352(3)	0.1859(3)	0.0514(7)
H(1A)	0.472(14)	0.262(5)	−0.070(6)	0.045
H(1B)	0.226(16)	0.257(7)	−0.115(7)	0.045
H(2A)	0.217(12)	0.196(6)	0.281(8)	0.042
H(2B)	0.030(13)	0.180(4)	0.219(7)	0.042
H(3A)	0.091(13)	0.358(5)	0.169(7)	0.043
H(3B)	0.333(13)	0.372(5)	0.185(7)	0.043
H(4A)	0.291(15)	0.088(6)	−0.033(9)	0.050
H(4B)	0.490(15)	0.096(5)	0.055(9)	0.050
H(1C)	0.219578	0.470002	0.056819	0.056
H(1D)	0.054428	0.402097	−0.008587	0.056
H(2C)	0.350584	0.410193	−0.108761	0.059
H(2D)	0.515847	0.394412	0.003908	0.059
H(3C)	0.290704	−0.014308	0.107412	0.062
H(3D)	0.072905	0.039773	0.078535	0.062
H(4C)	0.170862	0.043679	0.274845	0.061
H(4D)	0.408190	0.075190	0.262262	0.061
H(1E)	0.715(14)	0.229(7)	0.118(3)	0.048(18)
H(1F)	0.721(9)	0.2874(19)	0.210(6)	0.034(12)

* $U_{\text{e}q}=1/3\sum_{i}\sum_{j}U_{ij}a_{i}^{*}a_{j}^{*}({\vec{a}}_{i}{\vec{a}}_{j})$, for H atoms – *U*iso.

### Data S2: all-Union State Standard 12.1.044-89

*FIRE AND EXPLOSION HAZARD OF SUBSTANCES AND MATERIALS*.

*NOMENCLATURE OF INDICES AND METHODS OF THEIR DETERMINATION*

#### 4.7. The method of experimental determination of the ignition temperature of solids and materials

The method is carried out in the temperature range from 25 °C to 600 °C and is not used to test metal powders.

##### 4.7.1. Apparatus

**Figure 8:**
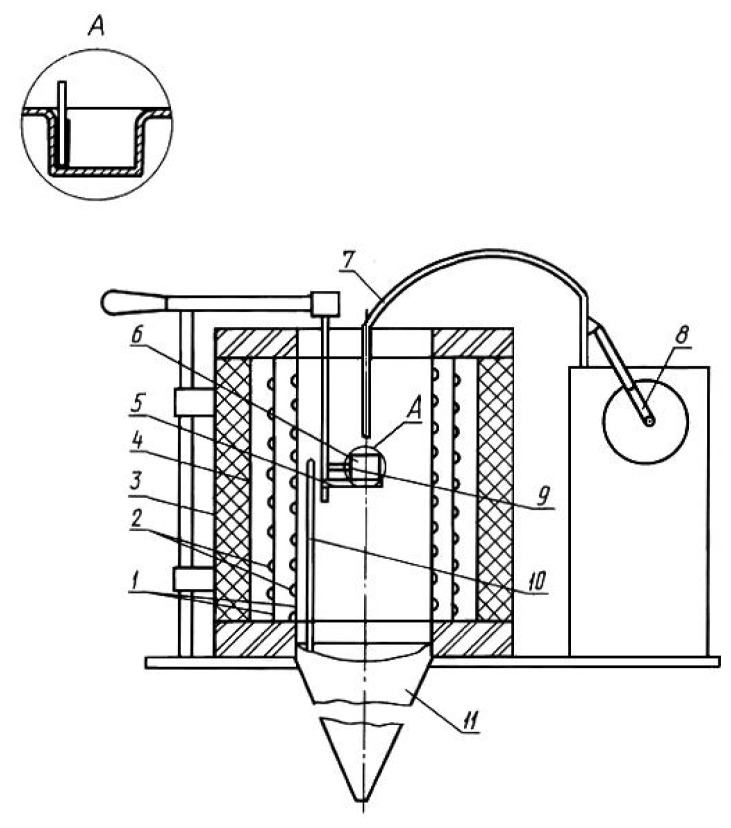
TF apparatus: 1 – glass cylinders; 2 – spiral electric heaters; 3 – heat-insulating material; 4 – steel screen; 5 – sample holder; 6 – container; 7 – gas burner; 8 – burner electric drive; 9, 10 – thermoelectric converters; 11 – laminator.

Scheme of TF apparatus for determination of the ignition and self-ignition temperatures is shown in Figure 8.

4.7.1.1. The TT apparatus, which is a vertical electric furnace with two coaxially located cylinders made of quartz glass. One of the cylinders with an inner diameter of (80 ± 3) mm and a height of 240 mm is a reaction chamber; the second cylinder of the same height has an inner diameter of (101 ± 3) mm. Spiral electric heaters with a total power of at least 2 kW are wound on the cylinders, which makes it possible to create a working zone temperature of 600 °C in no more than 40 minutes.

4.7.1.2. A container with a diameter of (45 ± 1) mm, a height of (18.0 ± 0.4) mm, made of steel mesh or steel sheet with a thickness of no more than 0.5 mm, is used to place a sample of the test substance (material). The container is placed in a holder ring with a diameter of (49.0 ± 0.6) mm. The holder is a metal tube made of heat-resistant steel with a ring welded at the bottom to accommodate the container.

4.7.1.3. A gas burner for ignition of a sample, which is a tube with an inner diameter of (6 ± 1) mm, is made of heat-resistant steel, sealed from below and has a hole on the side surface with a diameter of (0.8 ± 0.1) mm at a distance of (5.0 ± 0.5) mm from the sealed end.

4.7.1.4. A laminator made of sheet heat-resistant steel in the shape of a cone with an upper diameter of no more than 78 mm, a lower diameter of no more than 11 mm and a length of (500 ± 5) mm serves to supply a natural air flow into the reaction chamber.

4.7.1.5. Thermoelectric converters with a diameter of thermoelectrodes no more than 0.5 mm. Thermoelectric converter 9 is used to measure the temperature of the sample and is located in such a way as to ensure contact with the bottom and wall of the container (see Figure 8). Thermoelectric converter 10 serves to control and regulate the furnace temperature and is located inside the reaction chamber at a distance of (140 ± 5) mm from the upper edge of the chamber and (5 ± 1) mm from the chamber wall. The measurement error of the instruments regulating and recording the temperature should not exceed 0.5%.

4.7.1.6. Mirror for observing the sample inside the chamber.

4.7.1.7. Stopwatch with a measurement error of no more than 1 s.

4.7.1.8. Templates for determining the distance from the bottom edge of the torch to the surface of the sample and for centering the container inside the chamber.

4.7.1.9. Compressed air source for the burner with a flow rate of up to 40 L·h–1.

##### 4.7.2. Test preparation

4.7.2.1. For testing, prepare 10–15 samples of the test substance (material) with a mass of (3.0 ± 0.1) g. Samples of cellular materials should have a cylindrical shape with a diameter of (45 ± 1) mm. Film and sheet materials are collected in a stack with a diameter of (45 ± 1) mm, superimposing layers on top of each other until the specified weight is reached.

4.7.2.2. Before testing, the samples are conditioned in accordance with the requirements of GOST 12423 or material specifications. Samples should characterize the average properties of the test substance (material).

4.7.2.3. Depending on the volume of the sample, it is determined using templates and the position of the container inside the chamber and the distance between the gas burner and the surface of the sample are recorded.

4.7.2.4. The suitability of the installation for operation is checked against a standard substance – organic glass (GOST 10667), the ignition temperature of which is (265 ± 10) °C.

##### 4.7.3. Testing

4.7.3.1. The reaction chamber is heated to the temperature of the beginning of decomposition of the test substance (material) or to 300 °C.

4.7.3.2. By regulating the supply of gas and air to the burner, the flame of the gas burner is formed in the form of a wedge with a length of (10 ± 2) mm.

4.7.3.3. The holder with the container is removed from the chamber. The sample is placed in the container for a time not exceeding 15 s and it is introduced into the reaction chamber. The electric drive of the gas burner is switched on to the specified operating mode. The frequency of approaching the gas burner to the sample at a distance of (10 ± 1) mm from its surface should be (10 ± 2) s. Observe the sample in the working chamber using a mirror.

4.7.3.4. If the sample ignites at the test temperature, then the test is stopped, the burner is stopped in the “outside the oven” position, the container with the sample is removed from the chamber. Record the flash point in the report and the next test is carried out with a new sample at a lower temperature (for example, 50 °C less). If the sample does not ignite within 20 minutes or smoke emission completely stops before this time, then the test is stopped and the refusal is noted in the protocol. The test temperature is taken as the readings of the thermoelectric converter, which measures the sample temperature.

4.7.3.5. Using the method of successive approximations, using new samples and changing the test temperature, determine the minimum temperature of the sample at which, during the holding time in the furnace for no more than 20 minutes, the sample will ignite and burn for more than 5 s after removing the burner, and at a temperature of 10 °C less ignition is absent.

##### 4.7.4. Evaluation of the results

4.7.4.1. The arithmetic mean of two temperatures differing by no more than 10 °C is taken as the ignition temperature of the test substance (material), at one of which three samples are ignited, and at the other, three failures. The resulting temperature value is rounded to the nearest 5 °C.

4.7.4.2. The convergence of the method at a confidence level of 95% should not exceed 7 °C.

4.7.4.3. The reproducibility of the method at a confidence level of 95% should not exceed 20 °C.

4.7.4.4. Test conditions and results are recorded in the report.

## Figures and Tables

**Table 1:** Crystal data and experimental details for the single crystal of complex **1**.

Empirical formula	C_4_H_18_Cl_2_CuN_4_O
Formula mass (g mol^−1^)	272.66
Crystal system	Monoclinic
Space group	*P* 1 2_1_/*n* 1
Unit cell dimensions (Å, °)	
*a*	6.193(5)
*b*	15.214(7)
*c*	11.82(1)
b	98.5(1)
*V* (Å^3^), *Z*	1101(1), 4
Calculated density (g cm^−3^)	1.644
Absorption coefficient (mm^−1^)	2.435
*F*(000)	564
Crystal size (mm)	0.3**×**0.1**×**0.2
Crystal description	Dark-blue prism
Temperature (K)	295(2)
Wavelength (Å)	0.71073
2q_max_ (°)	61.89
Limiting indices	−8 ≤ *h* ≤ 8; −21 ≤ *k* ≤ 21; −17 ≤ *l* ≤ 12
Reflection collected	5306
Independent reflections	1462 [*R*_int_ = 0.0848]
Parameters/restraints	141/2
Goodness-of-fit on *F*^2^	1.033
Final *R* indices [*F* ≥ 4s(*F*)]	*R*_1_ = 0.0394, *wR*_2_ = 0.0705
*R* indices (all data)	0.0881
Weighing scheme [*w*]	[s^2^(F_o_^2^)+(0.0312*P*)^2^+0.0858*P*]^−1^ where *P* = (*F*_o_^2^+2*F*_c_^2^)/3

**Table 2:** Selected bond lengths and angles for complex **1**.

Bond	*d*, Å	Angle	ω,°
Cu–N(1)	1.996(3)	N(1)–Cu–N(2)	178.8(1)
Cu–N(2)	1.999(4)	N(1)–Cu–N(3)	85.3(1)
Cu–N(3)	2.029(3)	N(1)–Cu–N(4)	94.1(2)
Cu–N(4)	2.020(3)	N(2)–Cu–N(3)	95.6(1)
Cu–Cl(1)	2.831(3)	N(2)–Cu–N(4)	84.9(1)
Cu–O	2.659(4)	N(3)–Cu–N(4)	174.8(1)
		Cl(1)–Cu–N(1)	86.15(9)
O–H(1E)	0.87(5)	Cl(1)–Cu–N(2)	94.5(1)
O–H(1F)	0.87(4)	Cl(1)–Cu–N(3)	96.9(1)
		Cl(1)–Cu–N(4)	88.1(1)
N(1)–C(2)	1.469(6)	Cl(1)–Cu–O	171.91(9)
C(2)–C(1)	1.518(7)	O–Cu–N(1)	89.4(1)
C(1)–N(3)	1.475(6)	O–Cu–N(2)	89.8(1)
		O–Cu–N(3)	89.4(1)
N(2)–C(4)	1.465(6)	O–Cu–N(4)	89.8(1)
C(4)–C(3)	1.496(8)		
C(3)–N(4)	1.485(7)	N(1)–C(2)–C(1)	108.2(3)
		C(2)–C(1)–N(3)	107.9(4)
N–H	0.78(9)–1.07(9)	N(2)–C(4)–C(3)	108.0(4)
C–H	~0.97	C(4)–C(3)–N(4)	108.0(4)
